# Asialoglycoprotein receptor-mediated delivery of copper to hepatic tumors exerted inhibitory effect on tumor growth and progression

**DOI:** 10.1007/s10534-025-00772-9

**Published:** 2025-11-23

**Authors:** Maya P. Shetty, Priti Sule, Suresh D. Kulkarni, Pradip Chaudhari, Sanjay Bharati

**Affiliations:** 1 Department of Nuclear Medicine, Manipal College of Health Professions, Manipal Academy of Higher Education (MAHE), Manipal, Karnataka 576104 India; 2 Manipal Institute of Applied Physics, Manipal Academy of Higher Education (MAHE), Manipal, Karnataka 576104 India; 3 Comparative Oncology Program & Translational Preclinical Imaging and Radiotherapy Facility, Advanced Centre for Treatment Research and Education in Cancer (ACTREC), Kharghar, Mumbai 410210 India; 4 Homi Bhabha National Institute, BARC Training School Complex, Mumbai, 400094 India

**Keywords:** Copper, Targeted therapy, Copper overload, Hepatocellular carcinoma, Anti-angiogenic

## Abstract

Hepatocellular carcinoma (HCC) remains a major contributor to global cancer mortality and its rising incidence underscores the urgent need to explore novel therapeutic targets. Cancer is often characterized by dysregulated copper metabolism, which plays a crucial role in modulating tumor cell properties like cell proliferation, angiogenesis and metastasis. Therefore, exploiting their metabolic vulnerability using copper overload-based anticancer strategies has emerged as a novel approach. Despite the significant therapeutic potential of copper, its application in anticancer therapy has been limited due to systemic toxicity and non-target localization. In the present study we report targeted delivery of copper to the tumor site and its anticancer therapeutic potential of copper conjugated aminated arabinogalactan (Cu-aAG) in HCC. The synthesized compound was characterized using FT-IR, NMR, Mass spectroscopy and assessed for its anti-cancer therapeutic potential against the Wistar rat model of *N-nitrosodiethylamine-*induced hepatocellular carcinoma. The chemical characterization of Cu-aAG revealed successful copper complexation as evidenced by characteristic FT-IR peaks and elemental analysis showing 1.19% copper content. The involvement of amine and hydroxyl groups in the complexation was further confirmed by NMR and mass spectral analysis thus ensuring formation of stable, copper-centered co-ordination complexes. Cu-aAG treatment to tumor bearing Wistar rats significantly decreased tumor burden and tumor multiplicity (3.92 ± 2.9) as compared to untreated Tumor group (18.90 ± 3.02). Further, Cu-aAG treatment induced apoptotic cell death, cell cycle arrest, and inhibited angiogenesis. These findings highlight the potential of targeted delivery of copper overload-mediated anticancer therapy for inhibiting tumor growth and progression in HCC.

## Introduction

Hepatocellular carcinoma (HCC) is the predominant type of primary liver cancer and poses a significant global health concern. According to global cancer statistics, liver cancer accounts for more than 785,725 deaths annually worldwide, ranking as the third leading cause of cancer-related mortality (GLOBOCAN 2022). In India, HCC mortality has been reported at approximately 6.8 per 100,000 men and 5.1 per 100,000 women, and its incidence continues to rise due to increasing rates of viral hepatitis, metabolic syndrome, and alcohol-related liver injury (Konda & Vijayaraghavan 2025). The rising incidence and high mortality underscore the urgent need to explore novel therapeutic targets for the treatment of HCC.

Cancer cells often exhibit increased intracellular copper levels compared to their normal counterparts (Gupte and Mumper 2009). This is mainly because cancer cells actively rely on Cu-dependent enzymes essential for tumor cell proliferation and angiogenesis (Guan et al. 2023). Elevated intracellular copper levels enhance reactive oxygen species (ROS) generation, promote genomic instability, and modulate oncogenic signaling pathways, thereby facilitating tumor development and progression (Jian et al. 2020; Uriu-Adams and Keen 2005). However, exceeding copper levels beyond a certain threshold has been reported to be cytotoxic to cancer cells, resulting in inhibition of cancer progression (Wu et al. 2023). These findings have been well demonstrated in literature, suggesting excess copper triggers activation of cell death through mechanisms like apoptosis, ferroptosis, cuproptosis and paraptosis (Kawakami et al. 2008; Gandin et al. 2012; Ren et al. 2021; Liao et al. 2022; Tsvetkov et al. 2022).

Despite the significant therapeutic potential of copper, its application in anticancer therapy has been limited due to systemic toxicity and non-target localization (Pinho et al. 2021). Targeted therapy has now emerged as a promising new paradigm in cancer treatment. Among various targeting ligands, arabinogalactan (AG) is the most employed targeting agent for asialoglycoprotein receptor (ASGPR) mediated drug delivery to hepatoma cells (Ramírez-Cortés and Ménová et al. 2024). Its high binding affinity for ASGPR, which are overexpressed in hepatoma cells, ensures targeted delivery. In addition, its inherent anticancer property enhances the overall therapeutic effect. Therefore, in the present study, we have conjugated AG with copper for targeted delivery of copper to the tumor site and evaluated its anticancer therapeutic potential against HCC. To the best of our knowledge, this is the first study to demonstrate the in vivo anticancer therapeutic effect of an ASGPR-targeted copper complex, establishing a new approach for receptor-mediated, copper-based therapy in a rodent model of hepatocellular carcinoma.

## Materials and methods

### Chemicals and kits

Copper (II) chloride, Arabinogalactan and *N-nitrosodiethylamine* were procured from Sigma-Aldrich (St. Louis, USA). Antibodies for the detection of different protein expressions, including BAX (MA5-14,003), BCL-2 (MA5-11,757), Caspase-3 (MA1-91,637), Caspase-9 (PA5-16,358), PCNA (MA5-11,358), Ki-67 (MA5-14,520), VEGF (MA5-13,182), CD34 (PA5-85,917), were purchased from Thermo Fisher Scientific (Rockford, USA). AST (11,408,005), ALT (11,409,005), ALP (11,401,005), TC (51,403,002), HDL (5,101,001), LDL (11,415,003) and VLDL kits were purchased from AGAPPE Diagnostics, Kerala, India. In Situ Apoptosis Detection Kit (TACS-XL) was purchased from R&D Systems (Minneapolis, USA). All other reagents used in the study were of high purity grade and sourced from local Indian firms.

### Synthesis of copper-conjugated aminated arabinogalactan

Copper conjugated aminated arabinogalactan was synthesized using Kochetkov amination and metal ligand complexation method (Likhosherstov et al. 1986; Vetter and Gallop 1995; Mitić et al. 2011). 50 mg of arabinogalactan (AG) was dissolved in 5 ml of anhydrous dimethyl sulfoxide (DMSO) solution containing saturated ammonium carbonate. The reaction mixture was stirred continuously for 12 h at 45 °C in a closed vessel attached with a condensation unit. After 12 h the obtained solution was cooled and dialyzed against double-distilled water (DDW) for 48 h using a dialysis membrane with a molecular cutoff weight 3.5 KDa. The resulting complex was freeze-dried to yield a powdered lyophilized compound, aminated arabinogalactan. The aminated arabinogalactan was further reacted with copper ions to form a metal–ligand complex. Briefly, 161 mg of aminated arabinogalactan dissolved in 0.7 ml of distilled water was added to a solution containing 1.2 M NaOH. To this, a solution containing 0.17 mmol of CuCl_2_. 2H_2_O was added dropwise to the reaction mixture until the pH value of about 7 was reached. The obtained reactant mixture was then boiled for 7 min. The change in color of the reaction mixture from light blue to green indicated conjugation of copper to aminated arabinogalactan. After cooling, the reaction mixture was filtered, and the complex was precipitated by the addition of 2.5 mL of 96% ethanol. The precipitate was re-dissolved in 0.7 ml of distilled water and dialyzed for 1 h to remove unbound ions (Cl^–^, Na^+^, and Cu^2+^). Finally, the complex was dried for 3 h at 105 ֯C in a vacuum oven to yield pure copper with aminated arabinogalactan. Further, the obtained powdered compound was characterized for confirmation of copper-ligand complex formation.

### Characterization of copper-conjugated aminated arabinogalactan

#### Fourier Transform Infrared (FT-IR) Spectroscopy

The FT-IR spectrum of copper conjugated aminated arabinogalactan was obtained on FT-IR spectrophotometer (FTIR-8300 E, Shimadzu, Japan) in the frequency range 4000–400 cm^−1^. Background energy (percentage transmittance) was corrected using a blank KBr pellet. Each spectrum was baseline-adjusted using a polynomial function and then normalized to correct optical characteristics. The data obtained were analyzed for additions or alterations of chemical bonds.

#### Nuclear magnetic resonance (NMR) spectroscopy

^1^H-NMR and ^13^C-NMR were performed to define the structure and chemical composition of Cu-aAG. Freeze-dried powder of Cu-aAG was dissolved in D_2_O and read at 500 MHz by high resolution NMR spectrometer (ECX^500^ JEOL, US). Two-dimensional spectra were recorded using standard JEOL procedures. The NMR spectral data were then analyzed for changes at different proton and carbon positions.

#### Mass spectroscopy (MS)

The mass spectroscopic analysis was performed in a mass spectroscopy system LTQ XL linear ion-trap mass spectrometer (Ultimate 3000-LTQ XL, Thermo Fisher Scientific Corporation, Madison, WI, USA). The obtained data were evaluated for fragment separation and their relative abundance in the positive and negative ion modes.

### ‘2.3.4. Scanning electron microscopy with energy dispersive x-ray spectroscopy (SEM–EDX’)

SEM–EDX analysis was performed to obtain high-resolution surface morphology images of lyophilized Cu-aAG. Gold–palladium was sputtered on lyophilized Cu-aAG samples. The images were obtained at different magnifications, and elemental distribution was noted (ZEISS EVO 18, Germany).

### In vivo assessment of anticancer therapeutic potential of Cu-aAG in *N-nitrosodiethylamine-*induced hepatocellular carcinoma

#### HCC model and animal treatment

All animal experiments were conducted after getting approval from the Institutional Animal Ethics Committee *(IAEC/KMC/118/2022)*. Healthy male Wistar rats 250–300 g (8 weeks old) were procured from the Institutional Central Animal Facility, MAHE. These animals were housed in a controlled environmental condition (Temperature: 25 ± 1 °C, humidity: 65–80% and 12 h light/dark cycle). All animals were fed with a standard animal pellet diet and water ad libitum. The animals were acclimatized for 2 weeks prior to the start of the experiment. All animals were monitored for changes in body weight, diet intake, and water intake. Physiological observation of skin, fur, eyes, and physical behavior was done for all the animals throughout the study period.

Hepatocellular carcinoma model was developed using *N-nitrosodiethylamine* (NDEA) as per Jilkova et al. (Jilkova et al. 2018). Animals were randomly segregated into the following groups: Control, Cu-aAG, Tumor and Tumor + Cu-aAG (n = 6 each; total n = 24). Briefly, Tumor and Tumor + Cu-aAG group animals were administered with 50 mg/kg b.w. of NDEA intraperitoneally, once a week for a period of 14 weeks. The confirmation of tumor model development was done by assessing the glypican-3 (GPC3) levels in the serum. The animals with a twofold increase in GPC-3 levels as compared to the control group were considered HCC-positive animals. After the HCC model development period, Cu-aAG group and Tumor + Cu-aAG group animals were administered with 1 mg/kg b.w. of Cu-aAG intravenously, once a week for a period of 15 days. The control group animals did not receive any treatment. After the treatment period was completed, all animals were sacrificed for further investigations by administering an overdose of thiopentone sodium intraperitoneally.

#### Tumor statistics

The anti-tumor activity of Cu-aAG was evaluated in terms of tumor statistics, including hepatosomatic index, tumor size, tumor multiplicity and tumor dielectric parameter. The hepatosomatic index was calculated by dividing the liver weight by the total body weight of the animal. Tumor size was assessed by morphological examination, with tumors classified based on size as big tumors (≥ 3 mm) and small tumors (< 3 mm). The tumor multiplicity was calculated in terms of, total number of tumors counted in an animal to the total number of animals having tumors. The measurement of the dielectric parameter was performed using a four-pin electrode system (Shetty et al. 2020). Electrical parameters were noted in the frequency range (4 Hz-5 MHz). Thirteen or more data points (for each animal) were noted within 4 Hz to 5 MHz frequency range using LCR meter (Agilent E4980A Precision LCR Meter, USA). The tumors were then subsequently classified as moderate/high conductivity tumors based on their conductivity values.

#### Histopathological (H&E) assessment

Hepatic tissues/tumors were fixed in formalin for 48 h at room temperature. The samples were then subjected to dehydration through a series of graded ethanol (70–100%) treatment, followed by xylene wash, and paraffin wax embedding. Tissue sections of 5 μm thickness were cut and affixed to clean glass slides. Subsequently, the tissue sections were subjected to deparaffinization, rehydration and hematoxylin and eosin (H&E) staining. The stained tissue sections were then dehydrated, xylene-treated, and mounted with DPX mounting medium. The mounted sections were then assessed for histological changes under a light microscope (Labomed, Lx-300, USA).

#### Liver injury markers and lipid profile assessment

Liver injury markers alanine aminotransferase (ALT), aspartate aminotransferase (AST), alkaline phosphatase (ALP) were estimated from blood samples collected from each animal. In addition, the total lipid profile that includes Total Cholesterol (TC), Triglycerides (TG), high-density lipoprotein (HDL), low-density lipoprotein (LDL), and very-low-density lipoprotein (VLDL) was also quantified using the standard kit protocol (Agappe Diagnostics, Kerala, India).

#### Determination of type of cell death incurred by TUNEL assay

Immunohistostaining of hepatic tissues/tumors was performed to evaluate the type of cell death using TUNEL assay as described previously (Tambe et al. 2023). Briefly, paraffin-embedded tissue/tumor sections were deparaffinized, rehydrated and then incubated with proteinase K at 37 °C for 15 min. After the washing step, endogenous peroxidase activity was blocked using H_2_O_2_-methanol quenching solution for 5 min. Further, the slides were treated with 1X TdT labelling buffer followed by the addition of B-dNTP labelling reagent. The slides were then placed in a humidifying chamber (37 °C for 30 min). The slides were then allowed to react with a stopping buffer for 5 min to stop the reaction. Followed by a PBS wash, Anti-BrdU antibody solution was added and incubated in a humidifying chamber at 37 °C for 30 min. The slides were then rewashed in PBS-Tween 20 solution for 2 min and incubated in Strep-HRP solution for 10 min. Further, the DAB solution was added and incubated for 15 min. The slides were then washed, counterstained with methyl blue, dehydrated, and mounted in DPX. The slides were observed under a light microscope and evaluated for microscopic changes (Labomed, Lx-300, USA).

#### Quantitative assessment of expression of proteins related to apoptosis using ELISA

The level of apoptosis after treatment was assessed in terms of apoptotic markers (BAX, BCL-2, Caspase-3, and Caspase-9) using the ELISA technique as described by Tambe et al. (Tambe et al. 2025). In brief, antigens from liver tissues/tumors were extracted using Tris base-Sodium deoxycholate antigen extraction buffer. The extracted antigens were incubated in flat-bottom 96-well plates for 12 h at 4 °C. The plates were washed with PBST wash buffer, and bovine serum albumin (1%) was added as a blocking agent. Subsequently, 100 μL of primary BAX, BCl-2, caspase-3, and caspase-9 antibodies were added to the respective wells, and the plates were incubated at room temperature for 2 h. Further, post PBST wash, respective wells were treated with 100 μL of secondary antibodies and incubated for 1 h. Post incubation, the plates were washed and treated with 100 μL of 3,3′,5,5′-Tetramethylbenzidine (TMB) substrate for color development. This was followed by the addition of 50 µL (2 M sulfuric acid) stop solution. The plates were then read at 450 nm using a multiplate reader (BioTek Synergy H1, Agilent Technologies, USA), and the expression of respective markers was recorded.

#### Quantitative assessment of expression of proteins related to proliferation, differentiation, and angiogenesis using ELISA to evaluate the risk of recurrence and prognosis

The level of proliferation, differentiation, and angiogenesis after treatment was assessed in terms of proliferation marker (PCNA), differentiation marker (Ki-67), and angiogenesis markers (VEGF, CD34) as described previously (Qsee et al. 2022). In brief, antigens from liver tissues/tumors were extracted and incubated in flat-bottom 96-well plates for 12 h at 4 °C. The plates were washed and treated with a blocking agent (1% BSA). Further, the antigens were treated with primary (PCNA, Ki-67, VEGF, and CD34) antibodies and secondary antibodies (goat anti-mouse/anti-rabbit IgG secondary antibody) to the respective wells, and the plates were incubated for 2 h at room temperature. After the incubation period, washing, Tetramethylbenzidine (TMB) substrate addition, and stop solution treatment plates were read at 450 nm using a multiplate reader (BioTek Synergy H1, Agilent Technologies, USA), and expression of respective markers was noted.

#### Antioxidant defence status

Total ROS content was analyzed using a fluorescent probe DCFH-DA as described by Gupta et al. and expressed as arbitrary fluorescence unit (AFU) (Gupta et al. 2013). LPO level was analyzed as described by U et al. The rate of formation of malondialdehyde-thiobarbituric acid (MDA-TBA) chromophore was measured and expressed as nanomoles of MDA-TBA per milligram of protein per minute (Udupi et al. 2024). Glutathione (GSH) levels were measured by quantifying the total non-protein sulfhydryl groups as described by Weydert et al. and expressed as nanomoles of GSH per milligram of protein (Weydert and Cullen 2010). Glutathione reductase (GSH-R) activity was assessed using standard laboratory procedures (Shetty et al. 2019). Indirect assessment was performed by quantifying levels of NADPH and presented as nanomoles of NADPH utilized per minute per milligram of protein. Glutathione peroxidase (GSH-Px) activity was measured as described previously (Shetty et al. 2019). The reduction in NADPH concentration was measured and expressed as nanomoles of NADPH utilized per minute per mg of protein. Superoxide dismutase (SOD) activity was determined as described previously (U et al. 2022). The activity of SOD to neutralize superoxide radicals was quantified and expressed as international units per milligram of protein.

Total protein in the samples was estimated using the method described by Lowry et al. (Lowry et al. 1951). Briefly, under alkaline conditions, peptide nitrogen was allowed to interact with copper ions, triggering the reduction of phosphomolybdic acid to molybdenum blue. The change in absorbance was recorded at 620 nm with bovine serum albumin taken as a reference standard.

#### Copper content determination

The copper levels in hepatic tissues/tumors were determined using a colorimetric method as described by Abe et al. (Abe et al. 1989). Briefly, tissue samples were weighed and digested with 65% concentrated nitric acid at 100 °C for 1 h. The digested samples were cooled, and the residue was dissolved in 50% v/v hydrochloric acid. The copper concentrations were then measured using 3,5-DiBr-PAESA, a colorimetric reagent that forms a purple-colored complex with copper ions with maximum absorbance read at 580 nm (GENESYS 180, Thermo Scientific, Waltham, MA, USA). The concentration of copper was quantified from a standard curve prepared with known copper standards, normalized to total tissue protein. Results were expressed as µg Cu per mg protein.

#### Estimation of ceruloplasmin oxidase activity

Ceruloplasmin oxidase activity in hepatic tissues/tumor was quantified using *p*-phenylenediamine (PPD) as the method by Ravin with minor modifications (Ravin 1961). Briefly, tissue homogenates were prepared in ice-cold phosphate buffer and centrifuged at 12,000 × g for 10 min at 4 °C. The supernatant was incubated with 0.5 mM *p*-phenylenediamine in acetate buffer (pH 5.4) at 37 °C. After incubation for 30 min, the reaction was stopped by adding sodium azide (0.02%) to inhibit further oxidation. The absorbance of the oxidized product was measured at 530 nm (GENESYS 180, Thermo Scientific, Waltham, MA, USA) and expressed as micromoles of substrate oxidized per minute per milligram of protein. Results were normalized to total protein content.

#### Assessment of Cu/Zn superoxide dismutase activity

The enzymatic activity of Cu/Zn superoxide dismutase (SOD1) was measured as described earlier (Ghezzo-Schöneich et al. 2001). The enzyme's ability to catalyze the dismutation of superoxide radicals generated by the xanthine/xanthine oxidase was monitored. The inhibition of superoxide-driven oxidation of a chromogenic substrate was recorded and expressed as units per milligram of protein per minute.

#### Mitochondria isolation

Mitochondria isolation of hepatic tissues/tumors was performed as per the standard laboratory protocol (Qsee et al. 2022). Briefly, tissue samples were homogenized using pre-chilled mitochondria isolation buffer containing 0.25 M sucrose, 10 mM Tris–HCl and 0.5 mM EDTA (pH 7.4). The obtained homogenate was centrifuged at 2000 × g for 15 min. The obtained supernatant was re-centrifuged at 14,000 × g for 20 min. The resulting pellet containing isolated mitochondria was then fraction resuspended in suspension buffer (10 mM Tris–HCl, 0.25 M sucrose; pH 7.8) and used for subsequent analysis.

#### Assessment of cytochrome C oxidase activity

The activity of cytochrome C oxidase was estimated according to the method described by Tambe et al. Briefly, the hepatic mitochondrial fraction was incubated with 0.2% N-phenyl-p-phenylenediamine, 0.01% cytochrome C in 0.03 M phosphate buffer (pH 7.4). The change in absorbance at wavelength 550 nm, corresponding to the oxidation of cytochrome c, was recorded spectrophotometrically for 3 min. Cytochrome C oxidase activity was expressed as nmol/min/mg of mitochondrial protein.

### Statistical analysis

The statistical analysis was performed for the data expressed as mean ± SD. The Shapiro–Wilk test and Levene’s test were employed to evaluate normality and variance homogeneity, respectively. Intergroup comparisons were evaluated using one-way ANOVA followed by post-hoc test (Tukey’s HSD). Tumor multiplicity was assessed via Student’s t-test. A p ≤ 0.05 was considered statistically significant (Jamovi Desktop version 2.6.44).

## Results

### Physical appearance and characterization of synthesized copper-conjugated aminated arabinogalactan

The aminated arabinogalactan (aAG) obtained in the first step of synthesis appeared white in color. After copper conjugation, the final product, copper-conjugated aminated arabinogalactan (Cu-aAG) had a bluish-green appearance, which is a characteristic feature of copper complexes (Obtained yield 85%; melting point: 190–194 °C). Both the compounds arabinogalactan and copper-conjugated aminated arabinogalactan were characterized by FT-IR and NMR spectroscopy to confirm chemical modification and successful conjugation.

**Fig. 1:**
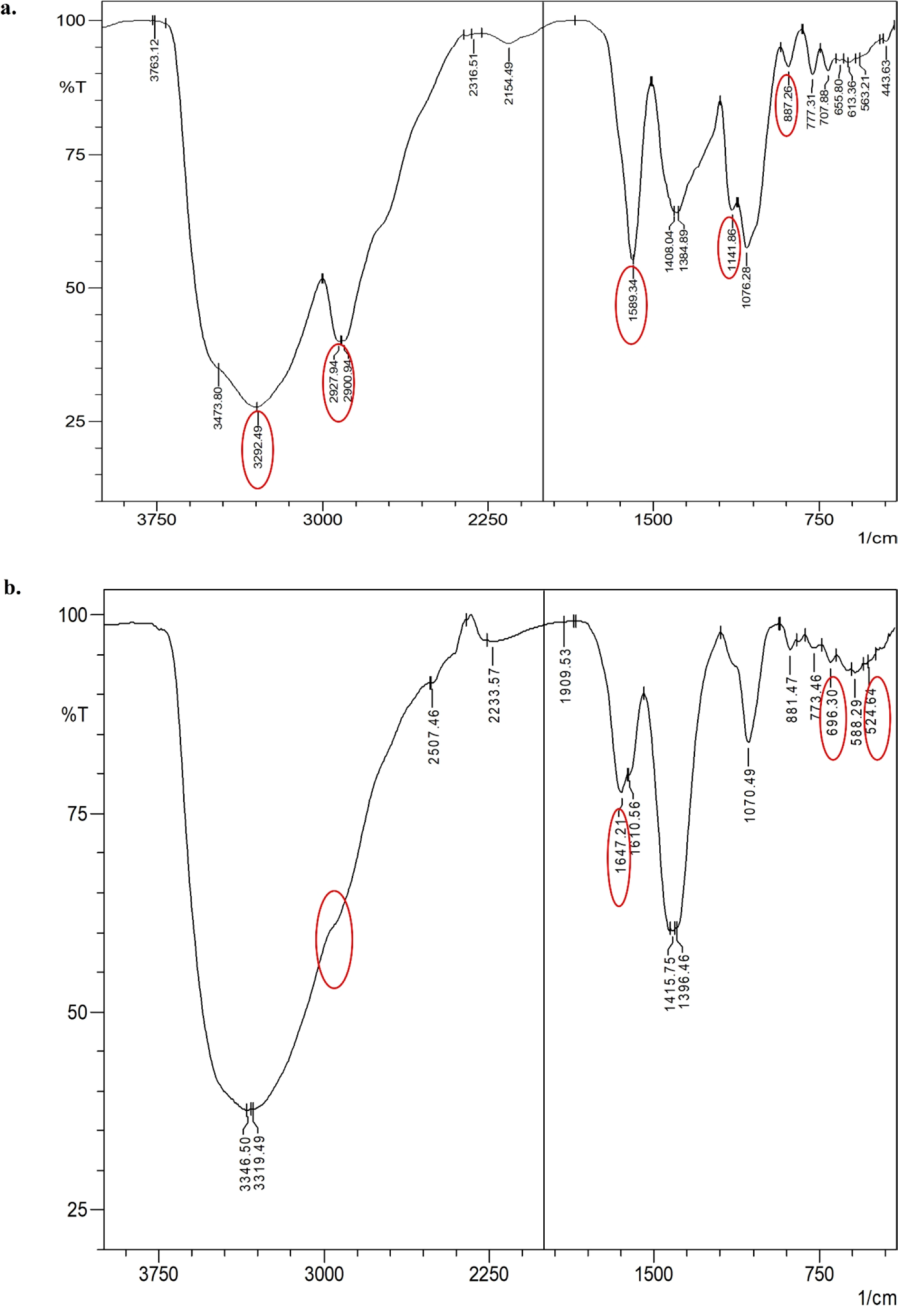
FT-IR spectrum of compounds. **a** FT-IR spectrum of arabinogalactan; **b** FT-IR spectrum of Cu-aAG

The FT-IR spectrum of native arabinogalactan (AG) (Fig. 1a) exhibited characteristic absorption bands at 3473 cm^−1^ and 3292 cm^−1^ corresponding with O–H stretching vibrations of typical carbohydrate polysaccharides. The peaks observed at 2927 cm^−1^ and 1384 cm^−1^ were attributed to C-H stretching and C-O bending vibration, respectively. The signal at 1076 cm^−1^ was indicative of C–O–C skeletal vibrations within the pyranose ring structure. The peaks at 887 cm^−1^ and 777 cm^−1^ were attributed to C–O–C linkages of β-glycosidic bonds. The results observed here for the FT-IR spectrum of AG were in concordance with the literature (Kuznetsov et al. 2018; Wu et al. 2020). The FT-IR spectrum of Cu-aAG (Fig. 1b) showed several distinct spectral changes confirming both amination and copper coordination. The shoulder observed at 2927 cm^−1^ is due to the -CH groups. The peaks between 696 cm^−1^ and 524 cm^−1^ were due to the presence of Cu–N and Cu–O stretching (Sportelli et al. 2017). The -NH bending was observed as two bands. The band at 1610 cm^−1^ is due to amine coordinated to copper, while the band at 1647 cm^−1^ is due to the free amine (Fig. 1b). Additionally, the peak observed at 3292 cm^−1^ for O–H stretching appeared to be decreased in intensity, indicating the involvement of hydroxyl groups in conjugation of copper with aminated arabinogalactan. Overall, these spectral modifications confirm the chemical transformation of AG into aminated and subsequently copper-conjugated derivatives.

**Fig. 2:**
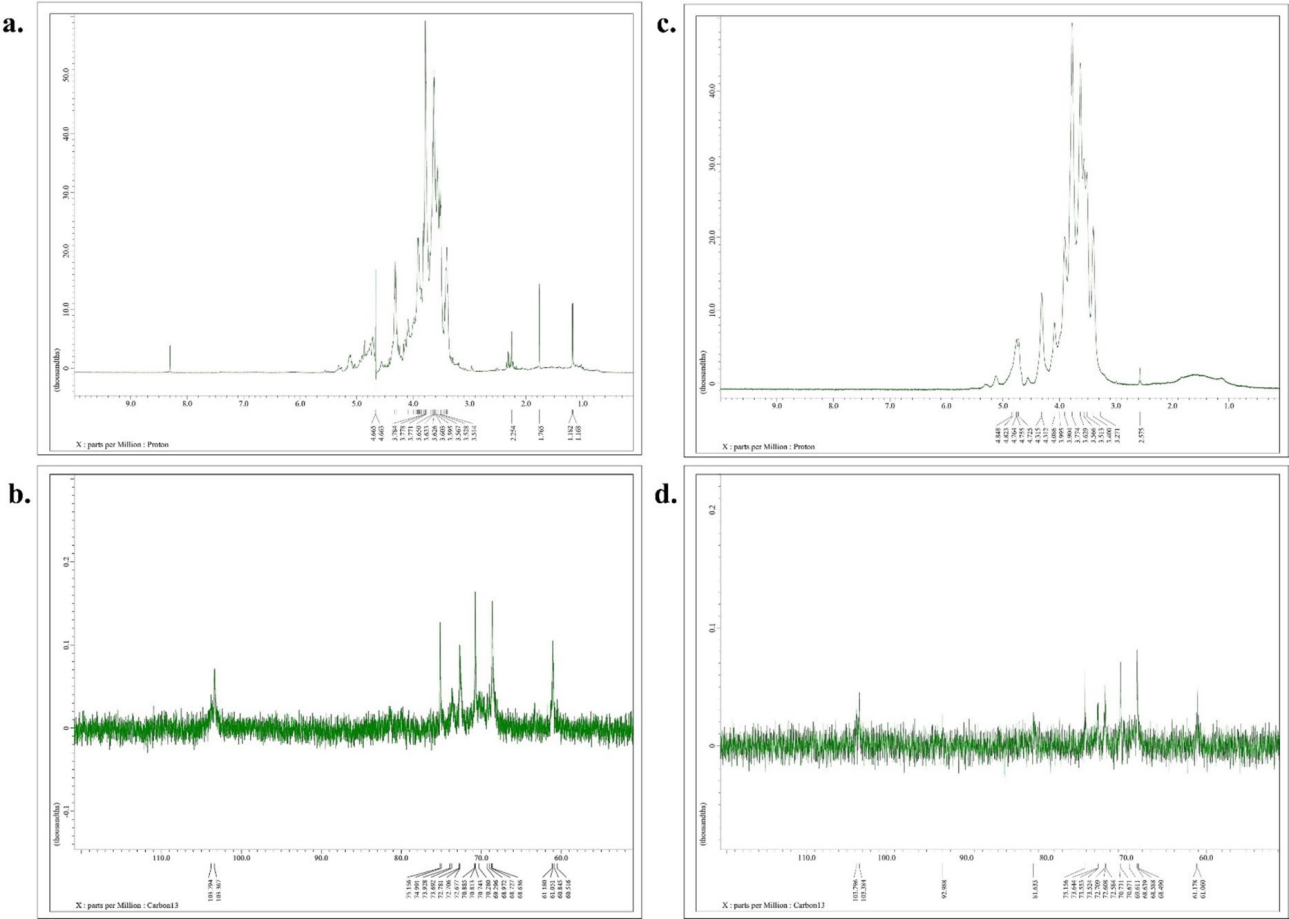
NMR spectrum of compounds. **a** & **b** ^1^H NMR and ^13^C NMR spectrum of arabinogalactan; **c** & **d** ^1^H NMR and ^13^C NMR spectrum of Cu-aAG

Further, the ^1^H NMR spectrum (Fig. 2a) of AG revealed distinct signals proton resonances corresponding to various positions in the galactose and arabinose residues. The signal at δ = 4.66 ppm was attributed to H1 and H3 of adjacent β-D-galactose units, involved in 1,3 glycosidic bonds. The signals at δ = 3.78 ppm corresponded to H6 of β-D-galactose involved in 3,6 glycosidic bonds. The peaks from δ = 3.51 to 3.77 ppm could be due to the resonating characteristic ring protons present at H-2 to H-5 β-D-galactopyranose. The signals at δ = 3.77 and 3.65 ppm might be due to protons at H-5 of α-L-arabinofuranose and β-D-glucuronic acid, respectively. The presence of peak at δ = 1.16 to 2.25 ppm attributed to CH_2_OH groups at C-6 and C-5 in β-D-galactopyranose and α-L-arabinofuranose (Speciale et al. 2022). ^13^C NMR spectrum (Fig. 2b) of AG demonstrated signals that corresponded to different carbon positions in the arabinogalactan chain. The peak at δ = 103.79 ppm was assigned to signals from C-1 position of galactose attached to C-6 position of another galactose unit linked via a glycosidic bond. The signals appearing between δ = 68.64 to 75.16 ppm were attributed to C-2, C-3, C-4, and C-5 of galactose. The signals at δ = 61.18 and 61.05 ppm were due to C-6 of galactose. Further, the peaks at δ = 70.74 and 61.05 ppm were assigned to C-4 and C-5 positions of α-L-arabinofuranose, respectively. The signal at δ = 60.85 ppm might be due to C-6 carbon (COOH) of glucuronic acid. The obtained NMR spectrum of AG is in accordance with the literature (Karácsonyi et al.^1984^; Do Nascimento et al. 2013; Rakhmanberdyeva et al. 2021).

The ^1^H NMR spectrum (Fig. 2c) of Cu-aAG revealed a new peak at δ = 2.58 ppm followed by spectral broadening at δ = 1.00 to 2.00 ppm, suggesting attachment of new functional groups such as amines (NH_2_) and copper ions (Cu (II)) by replacing hydroxyl groups of AG backbone. The appearance of new signals from δ = 4.09 to 4.85 ppm is due to the attachment of Cu (II) with AG through coordination with -NH groups (Al-Abdaly et al. 2015). The new peaks at δ = 3.77 to 3.99 ppm are due to involvement of C-6 in copper conjugation to aminated arabinogalactan. In ^13^C NMR spectrum of Cu-aAG (Fig. 2d), new signals appeared at δ = 81.63 and 92.99 ppm, indicating that modifications have taken place at C-4 position of aminated arabinose and 1,6 galactopyranosyl residues (Norberg et al. 2021). Additionally, the shift in signal at δ = 70.67 ppm adds emphasis that C-4 position of 1,3 galactopyranose is also involved in co-ordination complex formation between copper and arabinogalactan (Karácsonyi et al.^1984^). The upfield shift in signal at δ = 70.73 and 73.52 ppm indicated that the hydroxyl group at C-1 position of arabinose and galactose was substituted with amine groups during amination of AG. These observations collectively confirmed that copper ions coordinate primarily through the introduced amine and adjacent hydroxyl groups, forming a stable Cu (II)–ligand complex.

**Fig. 3:**
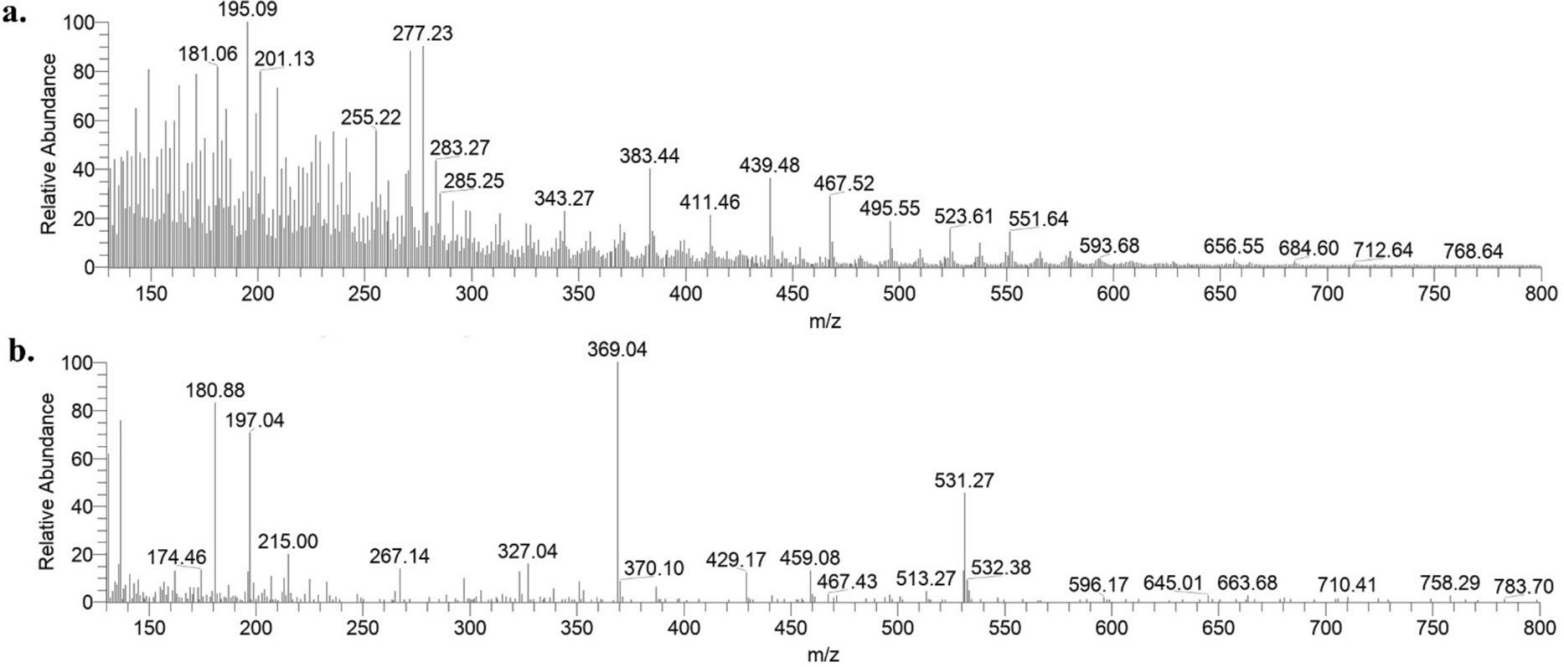
Mass spectra of copper conjugated aminated arabinogalactan. **a** Mass spectra of Cu-aAG in positive ion mode; **b** Mass spectra of Cu-aAG in negative ion mode

The Mass spectroscopy analysis of Cu-aAG (Fig. 3a and b) in the positive and negative ion mode indicated the separation of important fragments of Cu-aAG. In the positive ion mode, the separation of aminated galactose and glucuronic acid fragment was noted at a mass by charge ratio (m/z) of 181 and 195, respectively (Zhou et al. 2011). The separation of “Cu-C_6_H_12_O_6_” was noted at m/z of 255. The signal at m/z of 277 suggested the presence of copper conjugated aminated glucuronic acid (Pellei et al. 2022). The separation of aminated fragments of galactose was observed at m/z of 369. Further, the separation of “[Cu(C_6_H_13_NO_5_)OH]_2_” was noted at m/z of 531. Furthermore, the separation of the fragment at m/z of 383 was due to the presence of copper attached to “C_10_H_21_NO_9_”.

#### Arabinogalactan

White to beige powder; Melting point 240—245 °C; R (cm^−1^) (Fig. 1a): 3473 cm^−1^, 3292 cm^−1^, 2927 cm^−1^, 1384 cm^−1^,887 cm^−1^ and 777 cm^−1^; ^1^H NMR (500 MHz, D_2_O) δ ppm (Fig. 2a): 4.66 (d, 1H, *J* = 1.4 Hz), 3.78–3.51 (m, 5H), 2.25 (s, 5H), 1.16 (d, H *J* = 6.9); ^13^C NMR (500 MHz, D_2_O) δ ppm (Fig. 2c): 103.3 (t, 1 C J = 8.5 Hz), 75.2 (s, 5 C),73.7 (d, 5 C, J = 8.1 Hz),72.8–72.3 (m, 2 C); 70.9–69.9 (m, 6 C); 69.3–68.0 (m, 4 C).

#### Copper conjugated aminated arabinogalactan (Cu-aAG)

Light blue-green powder; Yield = 85%; Melting point 190 −194 °C; R (cm^−1^) (Fig. 1b): 3346 cm^−1^, 3319 cm^−1^,1647 cm^−1^,693 cm^−1^, 524 cm^−1^; ^1^H NMR (500 MHz, D_2_O) δ ppm (Fig. 2b): 4.884–4.755 (d, H6, *J* = 15.69 Hz), (s, H2) 4.315–4.312, (s, H2) 3.995–3.904, (s, H6) 2.575; ^13^C NMR (500 MHz, D_2_O) δ ppm (Fig. 2d): (t, C2, *J* = 5.9 Hz) 81.637, (t, C6, *J* = 14.9 Hz) 61.78–61.060).

**Fig. 4:**
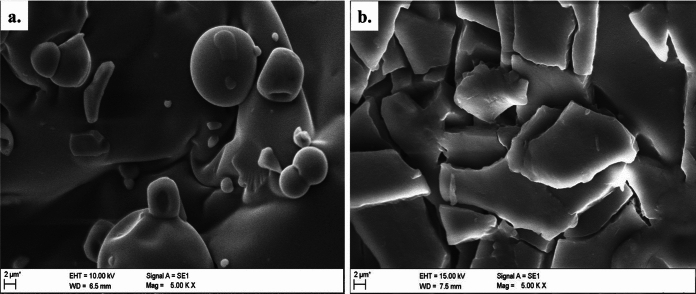
Surface morphology of Cu-aAG complex. **a** Scanning electron microscopy image of arabinogalactan smooth, globular, and amorphous morphology **b** Scanning electron microscopy image of Cu-aAG showing sheet-like flat surfaced structures indicating a significant alteration in surface morphology after copper-complexation

**Fig. 5:**
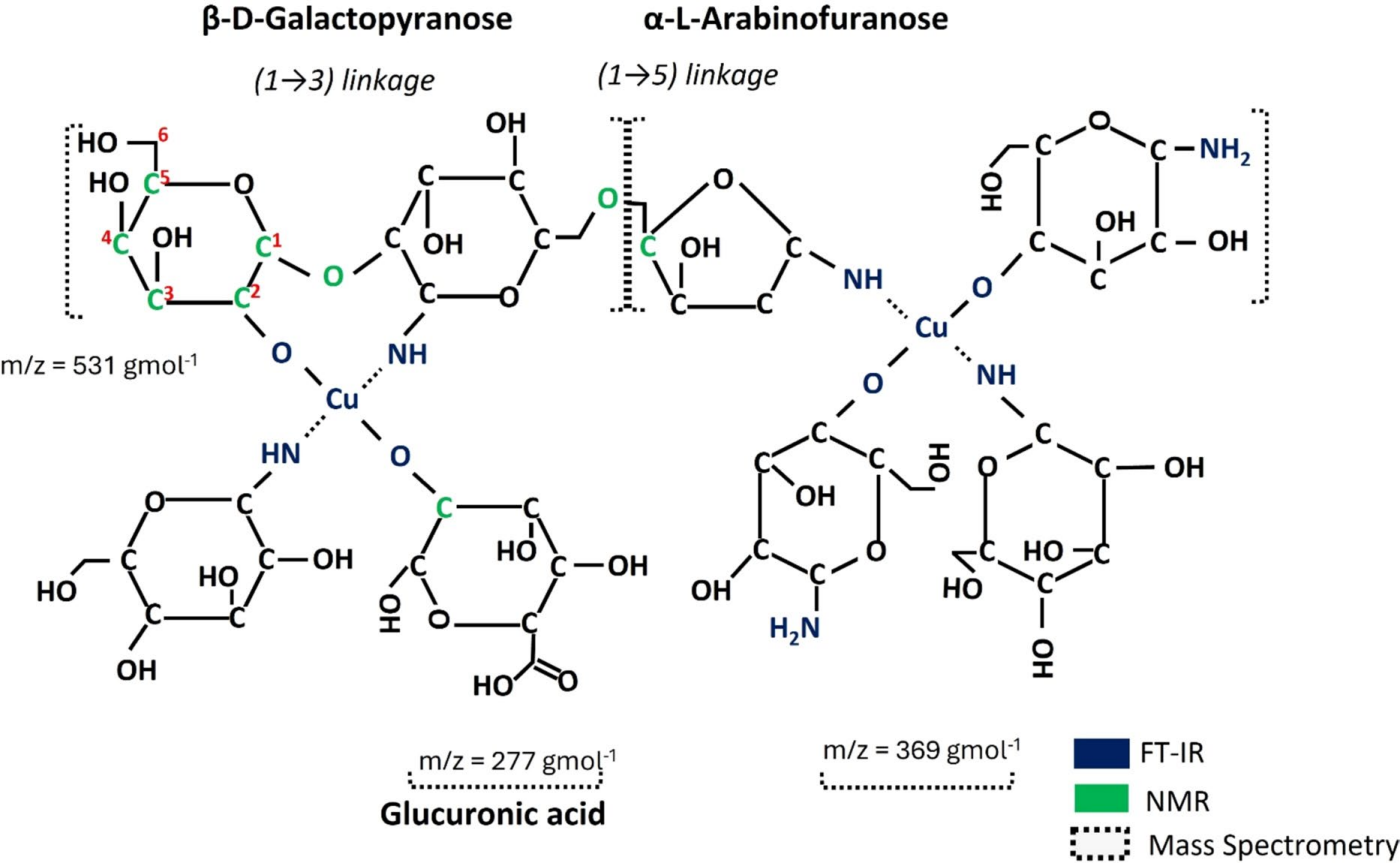
Structure of copper-conjugated aminated arabinogalactan. Probable structure of Cu-aAG with copper forming coordination complex with aminated arabinogalactan

The SEM–EDX analysis displayed morphological change from smooth spherical morphology of arabinogalactan (Fig. 4a) to a fragmented, layered, sheetlike structure after conjugation with copper (Fig. 4b). Further, elemental analysis of Cu-aAG revealed the presence of key elements (Weight percentage): C-52.27%, O-43.42%, N-3.12%, Cu-1.19% (Table 1). The probable structure of Cu-aAG, considering FT-IR, NMR, and mass spectral analysis, is shown in the figure (Fig. 5).

**Table 1:** EDX elemental composition of compounds

	Arabinogalactan (AG)		Copper conjugated aminated arabinogalactan (Cu-aAG)	
Elements	Weight %	Atomic %	Weight %	Atomic %
C	53.24	60.27	52.27	58.78
O	46.76	39.73	43.42	36.88
N	NIL	NIL	3.12	3.10
Cu	NIL	NIL	1.19	0.26
Total	100	–	100	–

Elemental composition of arabinogalactan compared with Cu-aAG in terms of weight percentage and atomic percentage

#### *Cu-aAG demonstrated *in vivo* anticancer therapeutic potential*

**Fig. 6:**
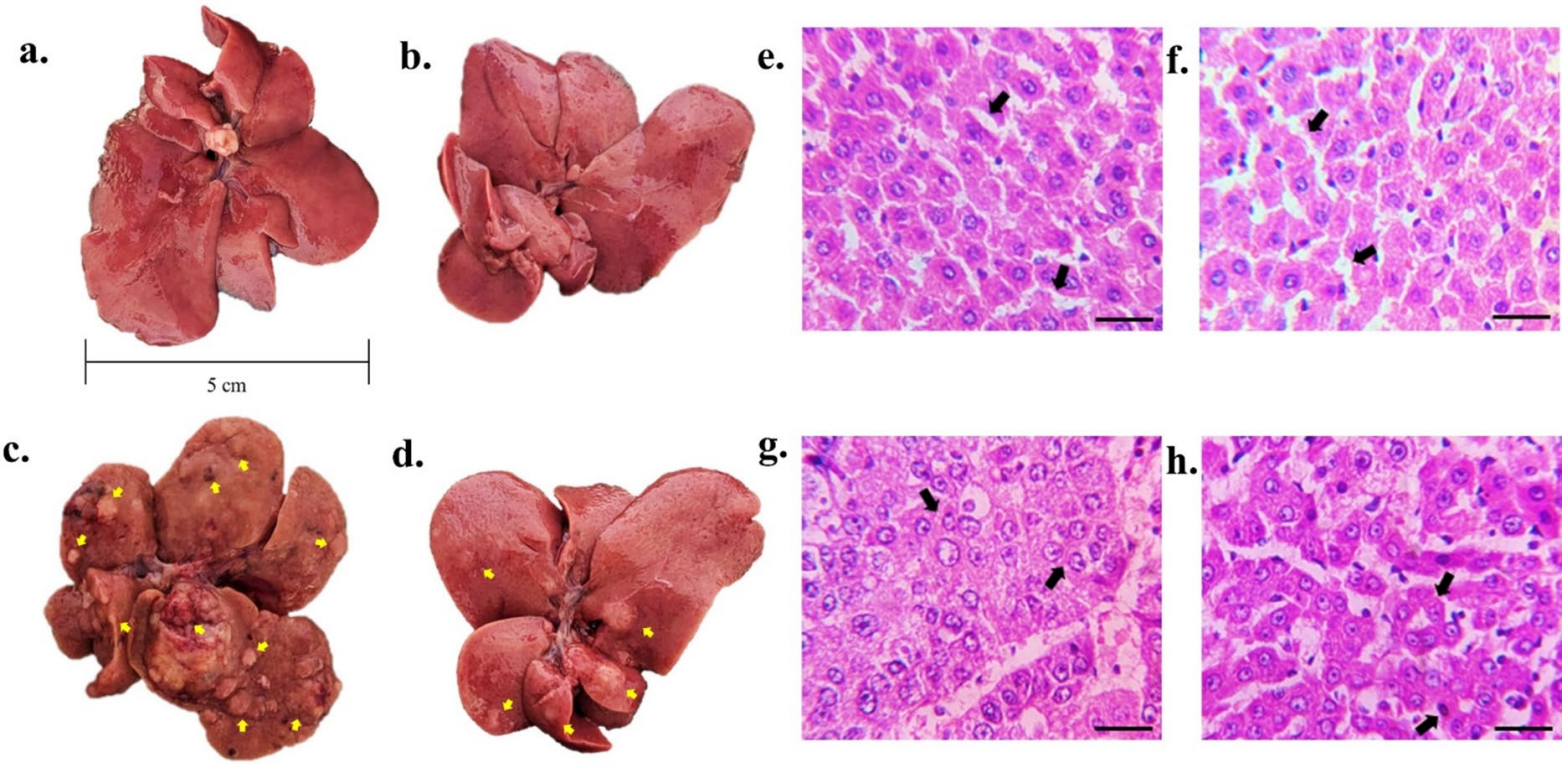
Gross morphology, H & E section of liver/liver tumors. **a** & **b** Control group and Cu-aAG group respectively with normal liver morphology and distinct liver lobes; **c** Tumor group with swollen liver lobes and large number of tumors (arrow); **d** Tumor + Cu-aAG group with altered liver morphology and comparatively small number of tumors (arrow); **e** & **f** Histology of control group and Cu-aAG group respectively with normal pattern of hepatocytes arrangement and presence of sinusoids (arrow); **g** Histology of Tumor group with poorly-differentiated HCC having solid pattern of growth, absence of sinusoids, and pleomorphism (arrow); **h** Histology of Tumor + Cu-aAG group with well-differentiated HCC having pseudoglandular or acinar formation (arrow) and abundant cytoplasm (Magnification: 400X with scale bar 50 μm)

The in vivo antitumor activity of Cu-aAG was assessed in *N-nitrosodiethylamine*-induced liver cancer. All animals, including the tumor-bearing animals, exhibited 100% survival with no mortality observed throughout the study period. The gross morphology of liver showed that control group and Cu-aAG groups animals had normal liver morphology with distinct liver lobes, whereas Tumor group animals had swollen liver lobes with large number of tumors and Tumor + Cu-aAG group animals had altered liver morphology with comparatively small number of tumors (Fig. 6). The histopathological analysis revealed most tumors were at poorly differentiated stage of HCC in the Tumor group. Tumor + Cu-aAG group showed well-differentiated HCC. The classical features of poorly differentiated HCC, such as a solid pattern of growth, absence of sinusoids, and pleomorphism, were observed in the Tumor group. Tumor + Cu-aAG group showed features of well-differentiated HCC, such as frequent pseudoglandular or acinar formation and abundant cytoplasm (Fig. 6g, h).

Further, the hepatosomatic index was significantly (*p* ≤ 0.05) decreased in Tumor + Cu-aAG group compared to Tumor group. After Cu-aAG treatment, a reduction in the total number of big and small tumors was observed in Tumor + Cu-aAG group compared to the Tumor group. In addition, tumor multiplicity was also significantly (*p* ≤ 0.05) decreased in Tumor + Cu-aAG group compared to Tumor group (Table 2). The animals in the Tumor group had 25% tumors with high electrical conductivity, whereas animals treated with Cu-aAG had less tumors with high electrical conductivity (Table 2).

**Table 2:** Effect of Cu-aAG on NDEA-induced HCC

Parameter	Control	Cu-aAG	Tumor	Tumor + Cu-aAG
Hepatosomatic Index	0.035 ± 0.005	0.035 ± 0.007	0.092 ± 0.033^*#^	0.045 ± 0.010^ϯ^
Big Tumors (≥ 3 mm)	–	–	275	61
Small Tumors (< 3 mm)	–	–	253	37
Tumor Multiplicity	–	–	18.90 ± 3.02	3.92 ± 1.80^ϯ^
Percentage of tumors showing high degree of conductivity	–	–	25.00%	15.00%

^*^: represents *p* ≤ 0.05 as compared to control group; ^#^: represents *p* ≤ 0.05 as compared to Cu-aAG group; ^ϯ^: represents *p* ≤ 0.05 as compared to Tumor group

#### Cu-aAG treatment protected against NDEA‑induced liver injury

The assessment of liver injury markers and lipid profile showed that Tumor group had significantly (p ≤ 0.05) increased AST, ALT, ALP, TC, TG, LDL and VLDL activity compared to the control group and Cu-aAG group. No significant change was observed in AST, ALT, ALP, TC, TG, LDL and VLDL activity in the Cu-aAG group compared to control group. Treatment of Cu-aAG to NDEA challenged animals in Tumor + Cu-aAG group significantly (p ≤ 0.05) decreased AST, ALT, ALP, TC, TG, LDL and VLDL activity compared to Tumor group (Table 3).

**Table 3:** Effect of Cu-aAG on liver injury markers and lipid profile

Parameter	Control	Cu-aAG	Tumor	Tumor + Cu-aAG
Aspartate aminotransferase (AST) (U/L)	95.01 ± 22.23	131.04 ± 30.17	287.53 ± 29.28^*#^	130.81 ± 28.85^ϯ^
Alanine aminotransferase (ALT) (U/L)	46.66 ± 6.56	69.28 ± 6.88	175.54 ± 20.68^*#^	121.53 ± 30.16^*#ϯ^
Alkaline phosphatase (ALP) (U/L)	321.78 ± 11.69	314.58 ± 10.90	630.08 ± 15.05^*#^	366.25 ± 35.37^*#ϯ^
Total cholesterol (TC) (mg/dL)	86.04 ± 1.16	86.11 ± 2.26	247.20 ± 3.10 ^*#^	156.12 ± 2.25 ^*#ϯ^
Triglycerides (TG) (mg/dL)	100.80 ± 3.45	115.12 ± 1.28	170.9 ± 4.66 ^*#^	130.61 ± 2.05 ^*#ϯ^
High-density lipoprotein (HDL) (mg/dL)	38.90 ± 0.66	37.521 ± 0.51	28.10 ± 0.88 ^*#^	31.30 ± 0.43 ^*#ϯ^
Low-density lipoprotein (LDL) (mg/dL)	26.94 ± 1.10	25.11 ± 0.91	192.72 ± 2.24^*#^	101.7 ± 1.54 ^*#ϯ^
Very low-density lipoprotein (VLDL) (mg/dL)	20.20 ± 0.23	21.12 ± 0.36	36.18 ± 0.54 ^*#^	28.65 ± 0.42 ^*#ϯ^

^*^: represents p ≤ 0.05 as compared to control group; ^#^: represents p ≤ 0.05 as compared to Cu-aAG group; ^ϯ^: represents p ≤ 0.05 as compared to Tumor group

####  Cu-aAG induced apoptotic cell death in liver tumors, possibly by suppressing the expression of anti-apoptotic protein and elevating the expression of pro-apoptotic protein.

**Fig. 7:**
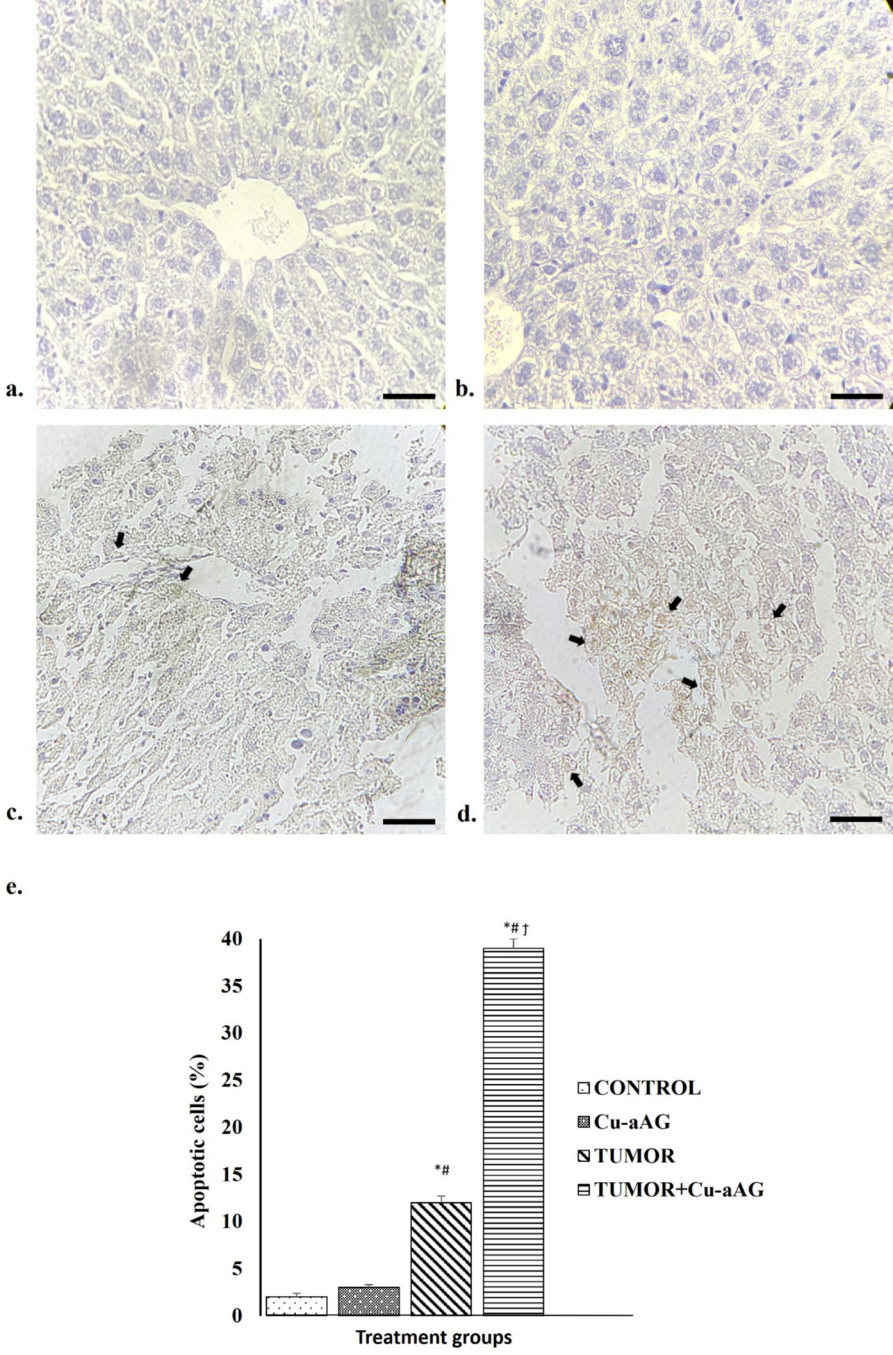
Cu-aAG induced apoptotic cell death in liver tissue/tumors. **a** & **b** represents control group and Cu-aAG groups respectively with normal hepatic cells. **c** Tumor group with the presence of darkly stained brown TUNEL-positive cells (arrow), indicating apoptosis. **d** Tumor + Cu-aAG group with increased number of TUNEL-positive cells compared to Tumor group (arrow). (Magnification: 400X with scale bar 50 μm). **e** Apoptotic cells percentage with a significant increase in the percent apoptotic cells in Tumor + Cu-aAG group compared to Tumor group. (^*^: represents p ≤ 0.05 when compared with the control group; ^#^: represents p ≤ 0.05 when compared with the Cu-aAG group; ^ϯ^: represents p ≤ 0.05 when compared with the Tumor group)

TUNEL-stained sections of hepatic tissue from various treatment groups were examined to analyze the type of cell death. The control and Cu-aAG groups showed normal hepatocytes with minimal TUNEL-positive apoptotic cells (Fig. 7a, b). In contrast, Tumor group and Tumor + Cu-aAG group displayed TUNEL-positive apoptotic cells with a brown coloration (indicated by black arrows) (Fig. 7c, d). The overall percentage of TUNEL-positive apoptotic cells in Tumor + Cu-aAG group was significantly (p ≤ 0.05) compared to Tumor group (Fig. 7e).

Further, on assessing the expression of proteins related to apoptosis it was found that the level of anti-apoptotic protein BCL-2 was significantly (p ≤ 0.05) decreased in Tumor + Cu-aAG group as compared to Tumor group. Conversely, there was a significant (p ≤ 0.05) increase in the levels of pro-apoptotic proteins BAX, Caspase-3, and Caspase-9 in Tumor + Cu-aAG group as compared to Tumor group (Table 4).

**Table 4:** Effect of Cu-aAG on apoptosis markers

Parameter (OD/mg tissue protein)	Control	Cu-aAG	Tumor	Tumor + Cu-aAG
BAX	0.403 ± 0.053	0.402 ± 0.033	0.29 ± 0.029^*#^	0.396 ± 0.043^ϯ^
BCL-2	0.301 ± 0.065	0.303 ± 0.069	0.427 ± 0.060^*#^	0.323 ± 0.069^ϯ^
Caspase-3	0.241 ± 0.015	0.231 ± 0.010	0.206 ± 0.006^*#^	0.229 ± 0.003^ϯ^
Caspase-9	0.292 ± 0.030	0.288 ± 0.033	0.209 ± 0.022^*#^	0.279 ± 0.042^ϯ^

^*^: represents p ≤ 0.05 as compared to control group; ^#^: represents p ≤ 0.05 as compared to Cu-aAG group; ^ϯ^: represents p ≤ 0.05 as compared to Tumor group

#### Cu-aAG suppressed the expression of proteins related to proliferation, differentiation, and angiogenesis in liver tumors

On assessing the expression of PCNA, Ki-67, VEGF, and CD34 it was found that the levels of these proteins were significantly (p ≤ 0.05) elevated in Tumor group as compared to control group. Remarkably, administration of Cu-aAG, led to a significantly (p ≤ 0.05) decreased level of the proteins in Tumor + Cu-aAG group compared to the Tumor group (Table 5).

**Table 5:** Effect of Cu-aAG on proliferation, differentiation, and angiogenesis markers

Parameter (OD/mg tissue protein)	Control	Cu-aAG	Tumor	Tumor + Cu-aAG
PCNA	0.789 ± 0.045	0.821 ± 0.013	0.925 ± 0.03^*#^	0.837 ± 0.009^ϯ^
Ki-67	0.773 ± 0.02	0.756 ± 0.04	0.878 ± 0.004^*#^	0.824 ± 0.015^ϯ^
VEGF	0.775 ± 0.005	0.78 ± 0.02	0.872 ± 0.003^*#^	0.818 ± 0.047^ϯ^
CD34	0.768 ± 0.018	0.749 ± 0.023	0.910 ± 0.083^*#^	0.786 ± 0.078^ϯ^

^*^: represents p ≤ 0.05 as compared to control group; ^#^: represents p ≤ 0.05 as compared to Cu-aAG group; ^ϯ^: represents p ≤ 0.05 as compared to Tumor group

#### Cu-aAG effectively modulated the antioxidant defence system of liver tumors

Cu-aAG effectively modulated tumor’s antioxidant defence status (Table 6). A significantly (p ≤ 0.05) depressed ROS and LPO was observed in Tumor + Cu-aAG group compared to Tumor group. Additionally, a significant (p ≤ 0.05) improvement in the levels of GSH, GSH-R, GSH-GPx, and SOD were observed in Cu-aAG treated Tumor + Cu-aAG group compared to Tumor group. Cu-aAG alone treatment had no significant effect on any of the antioxidant enzymes, lipid peroxidation, and reactive oxygen species compared to control group (Table 6).

**Table 6:** Effect of Cu-aAG on an antioxidant defence system in liver tissue/tumors

Parameter	Control	Cu-aAG	Tumor	Tumor + Cu-aAG
ROS (AFU)	638.975 ± 27.656	635.100 ± 21.936	968.600 ± 30.304^*#^	767.550 ± 20.757^*#ϯ^
LPO (nmole/min/mg protein)	9.841 ± 1.858	9.586 ± 0.998	18.286 ± 1.526^*#^	12.632 ± 0.980^*#ϯ^
GSH (nmole/mg protein)	7.313 ± 1.090	7.235 ± 0.627	2.736 ± 0.226^*#^	4.295 ± 1.273^*#ϯ^
GSH-R (nmole/min/mg protein)	0.647 ± 0.028	0.637 ± 0.022	0.341 ± 0.038^*#^	0.493 ± 0.046^*#ϯ^
GSH-Px (nmole/min/mg protein)	1.901 ± 0.145	1.857 ± 0.237	0.787 ± 0.292^*#^	1.276 ± 0.080^*#ϯ^
SOD (IU/mg protein)	20.976 ± 2.088	20.958 ± 0.504	12.232 ± 0.373^*#^	16.948 ± 0.663^*#ϯ^

^*^: represents p ≤ 0.05 as compared to control group; ^#^: represents p ≤ 0.05 as compared to Cu-aAG group; ^ϯ^: represents p ≤ 0.05 as compared to Tumor group

#### Cu-aAG demonstrated increased copper accumulation in hepatic tumors

**Fig. 8:**
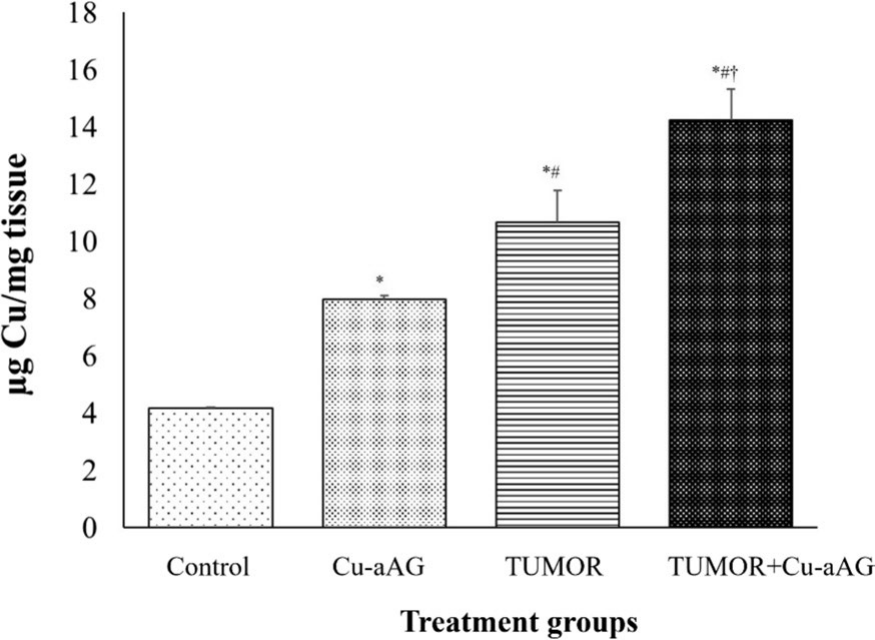
Cu-aAG levels in different treatment groups. The graph represents the changes in intracellular copper levels in different treatment groups (^*^: represents p ≤ 0.05 when compared with the control group; ^#^: represents p ≤ 0.05 when compared with the Cu-aAG group; ^ϯ^: represents p ≤ 0.05 when compared with the Tumor group)

The quantitative estimation of copper levels in hepatic tissue showed that the normal control group had a minimum copper concentration of 4.18 µg Cu/mg protein. Cu-aAG administration to the normal control group (Cu-aAG) elevated hepatic copper levels to 7.89 µg Cu/mg protein. Further, Tumor group (10.6 µg Cu/mg protein) showed significant (p ≤ 0.05) increase in copper levels as compared to control and Cu-aAG. However, Tumor+Cu-aAG group demonstrated significantly (p ≤ 0.05) increased copper levels (14.22 µg Cu/mg protein) as compared to other groups indicating effective copper overloading in hepatic tumors (Fig. 8).

#### Cu-aAG effectively modulated copper metabolism

Cu-aAG treatment significantly (p ≤ 0.05) increased the enzymatic activities of cytochrome c oxidase and Cu/Zn superoxide dismutase as compared to Tumor group. In addition, tissue ceruloplasmin oxidase activity was also significantly (p ≤ 0.05) increased in Tumor + Cu-aAG group as compared to Tumor group (Table 7).

**Table 7:** Effect of Cu-aAG on copper metabolism in liver tissues/tumors

Parameter	Control	Cu-aAG	Tumor	Tumor + Cu-aAG
Ceruloplasmin oxidase (nmol/min/mg)	22.18 ± 0.094	22.99 ± 0.06	35.44 ± 0.102^*#^	46.17 ± 0.104^*#ϯ^
Cu/Zn superoxide dismutase (U/mg protein)	1.45 ± 0.098	1.32 ± 0.030	0.75 ± 0.041^*#^	0.93 ± 0.046^*#ϯ^
Cytochrome C oxidase (nmole NADH oxidized/min/mg protein)	0.75 ± 0.004	0.709 ± 0.005	0.40 ± 0.006^*#^	0.54 ± 0.001^*#ϯ^

^*^: represents p ≤ 0.05 as compared to control group; ^#^: represents p ≤ 0.05 as compared to Cu-aAG group; ^ϯ^: represents p ≤ 0.05 as compared to Tumor group

## Discussion

Copper is a fundamental trace element required for the proper functioning of various cellular processes (Xue et al. 2023). Its role as a catalytic co-factor is indispensable in numerous redox-active enzymes that participate in mitochondrial respiration, antioxidant defense and cellular signaling (Ling et al. 2025). The disruption of copper homeostasis impairs the COX activity leading to decreased ATP production and elevated generation of reactive oxygen species (Vo et al. 2024). During HCC, dysregulated copper metabolism has been increasingly recognized as a contributing factor for tumor growth and progression (Tsang et al. 2020; Shan et al. 2025). Notably, the crucial role of copper in liver physiology makes copper a suitable target for the treatment of hepatocellular carcinoma (HCC) (Zhou et al. 2023). However, limitations such as systemic toxicity and non-target localization have hindered its widespread application in anticancer therapy (Noh et al. 2024). Targeted therapy has been considered a promising strategy to overcome the therapeutic limitations of copper (Abdolmaleki et al. 2024). Therefore, in the present study, we conjugated copper with arabinogalactan for targeted delivery of copper to the tumor site and evaluated its anticancer therapeutic potential in the treatment of HCC.

Copper-conjugated aminated arabinogalactan was synthesized using Kochetkov amination followed by a co-precipitation method which is well well-established method for metal–ligand complexation (Likhosherstov et al. 1986; Mitić et al. 2011). It is well documented that metal ions form strong coordination bonds with free amine groups due to their high electron-donating ability (Gritsch et al. 2018; Podjed et al. 2022). However, native arabinogalactan (AG) lacks amine groups and instead contains only free hydroxyl groups, which exhibit relatively weak binding affinity with metal ions (Al-Sogair et al. 2011; Wu et al. 2020). This was a major limitation for the direct conjugation of AG with metal ions. Therefore, in the present study, arabinogalactan was first aminated to introduce primary amine groups onto the AG backbone to facilitate the formation of strong and stable coordination complexes with copper ions. Consequently, a bluish green coloured precipitate (Cu-aAG) with unreacted components in the supernatant was obtained. The precipitated Cu-aAG complex was then subsequently washed with ethanol and subjected to dialysis to ensure removal of any unreacted, free or unbound ion. The successful complexation of copper with aminated arabinogalactan was indicated by the appearance of newer characteristic absorption peaks corresponding -NH_2_ (1647 cm^−1^), Cu–N (696 cm^−1^) and Cu–O (524 cm^−1^) stretching vibrations in the FT-IR spectrum. Further, the presence of copper (1.19%) in elemental analysis of the final synthesized product confirmed successful conjugation of copper to the aminated arabinogalactan. The involvement of hydroxyl groups and amine groups in metal ligand complexation was further confirmed through ^1^H NMR and ^13^C NMR spectral analysis. The appearance of new peaks at δ = 2.58 ppm, 4.09 to 4.8 ppm in ^1^H NMR and δ = 81.63 and 92.99 ppm in ^13^C NMR confirmed attachment of amine groups and copper ions to AG backbone (Norberg et al. 2021). The mass spectral analysis further demonstrated separation of different copper-conjugated aminated arabinogalactan fragments that included copper-galactose, copper-arabinose and copper-glucuronic acid. Overall, these results indicated that Cu-aAG was formed by involving amine groups at C-1 position and hydroxyl groups at C-2 and C-4 position of AG resulting in metal-centred cage-like structure forming coordination complexes.

Following successful conjugation of aminated AG with copper, the in vivo anticancer potential of Cu-aAG was evaluated in the *NDEA*-induced HCC model. The weekly administration of NDEA for a period of 14 weeks resulted in the development of HCC in the Wistar rat model. The development of HCC model was confirmed through the assessment of serum glypican-3, an early serum marker (Udupi et al. 2024). Animals exhibiting twofold or more increase in serum glypican-3 were then randomly segregated into Tumor and Tumor + Cu-aAG groups. After 15 days of Cu-aAG treatment, the total number of liver tumors and tumor multiplicity in Tumor + Cu-aAG treated group were significantly decreased as compared to the untreated Tumor group. The total percent tumor inhibition of 83% further indicated the effective anticancer activity of this complex. These results were in accordance with a previous study conducted by Wang et al. where copper complex (trinuclear-thiophene-2-carboxaldehyde-thiosemicarbazone-Cu(I) bromide) demonstrated a significant tumor inhibition effect in the BALB/c xenograft model of bladder cancer (Wang et al. 2024). Further, the results of tumor inhibition by Cu-aAG were equivalent to or better than the literature reported tumor inhibition values by the standard chemotherapeutic drug doxorubicin (Hassan et al. 2021; Di et al. 2008; Fiume et al. 2005).

The tumor inhibition effect demonstrated by Cu-aAG could be attributed to different types of cell deaths induced by copper overload in the cancer cells. This included apoptosis, cuproptosis, autophagy, necrosis, ferroptosis, and pyroptosis (Chen et al. 2023). In the present study, the induction of apoptosis was clearly evidenced by significantly increased expression of pro-apoptotic proteins like BAX, Caspase-3 and Caspase-9 along with the presence of TUNEL-positive cells in Cu-aAG treated group as compared to the untreated tumor group. Although in the present study we have only evaluated apoptosis-induced cell death, it is likely that other forms of cell death, such as cuproptosis, might have also played an active role in copper-induced cell death.

Cuproptosis, a type of regulated cell death triggered by excessive intracellular copper accumulation. Elevated copper levels beyond cellular threshold disrupt copper homeostasis, perturb key metabolic processes, thereby triggering cuproptosis-induced cell death. This was indicated by copper quantification analysis of treated groups, which showed that Cu-aAG treatment significantly increased intracellular copper levels and altered copper metabolism of tumor cells.

In addition to its role in promoting cell death, copper overload therapy is known to interfere with key hallmark characteristics of tumor progression, i.e. cell proliferation and angiogenesis. This was clearly evidenced in our study, where Cu-aAG treated group demonstrated significantly decreased expression of cell proliferation proteins like PCNA and Ki-67 as compared to the untreated tumor group. This is likely attributed to the ability of excess intracellular copper ions to disrupt redox homeostasis leading to oxidative stress-induced DNA damage that triggers cell cycle arrest via p53-p21 pathway and inhibition of cyclin-dependent kinases, resulting in suppression of cell proliferation proteins like PCNA and Ki-67 (Ji et al. 2023). Similar findings were observed in a study conducted by Abdolmaleki et al. on BEL-7404 cells treated with copper (II) complexes with pyridine-2,6-dicarboxylate, where copper induced increased ROS generation resulting in arrest at G_2_/M phase of the cell cycle (Abdolmaleki et al. 2023). In another study conducted by Zheng et al. also demonstrated that the copper complex showed antiproliferative activity against TNBC cancer cells. Additionally, these copper complexes not only inhibited proliferation but also decreased the expression of angiogenic proteins like VEGF and CD34 (Zheng et al. 2023). Our results were in accordance with these findings, where Cu-aAG treated group demonstrated significantly decreased VEGF and CD34 expressions as compared to untreated tumor group. This ability of copper to inhibit angiogenesis might be attributed to copper overload-induced inactivation of hypoxia-inducible factor 1-alpha (HIF-1α) that subsequently impairs VEGF-mediated signaling pathway, resulting in inhibition of neovascularization and endothelial cell proliferation (Zheng et al. 2023). Further, evidence in literature has also suggested that copper overload interferes with PI3K/AKT and MAPK/ERK pathways, contributing to marked inhibition of tumor cell proliferation and angiogenesis (Abdolmaleki et al. 2024). Overall, these results suggested that Cu-aAG treatment not only promoted apoptosis but also inhibited cell proliferation and angiogenesis.

In conclusion, it can be suggested that targeted copper overload via ASGPR-mediated copper delivery can be a novel anticancer therapeutic strategy for the inhibition of hepatic tumor growth and progression. This was clearly demonstrated in our results, where Cu-aAG treatment showed significantly decreased tumor burden and restored histoarchitecture compared to the untreated tumor group. Furthermore, its ability to induce cell cycle arrest, inhibit angiogenesis and promote regulated cell death underscores its potential as an effective anticancer agent against HCC. However, the underlying mechanism of Cu-aAG’s therapeutic effect remains to be fully elucidated. Although apoptosis-mediated cell death was clearly observed, the possible contribution of cuproptosis or other regulated cell death pathways could not be directly confirmed due to the lack of experimental evidence, which represents one of the limitations of this study. Therefore, future studies incorporating cuproptosis-related markers such as ferredoxin 1 (FDX1), lipoic acid synthetase (LIAS), lipoyltransferase1 (LIPT1), and dihydrolipoamide S-acetyltransferase (DLAT), along with a detailed examination of intracellular copper distribution and molecular pathways, would add further valuable insights into the mechanistic action of Cu-aAG against HCC.

## Data Availability

Data are available and can be provided upon a request to the corresponding author.
